# The complete chloroplast genome and phylogenetic analysis of *Jasminum lanceolaria* Roxb. (Oleaceae)

**DOI:** 10.1080/23802359.2026.2622811

**Published:** 2026-02-04

**Authors:** Xianglan Liang, Guangfu Tian, Song Guo, Chang Liu

**Affiliations:** a School of Chemistry and Chemical Engineering, Guangdong Pharmaceutical University, Guangzhou, P.R. China; b Institute of Medicinal Plant Development, Chinese Academy of Medical Sciences & Peking Union Medical College, Beijing, P.R. China; c College of Smart Agriculture (College of Internet of Things Engineering), Guangxi Science and Technology Normal University, Laibin, P.R. China; d School of Traditional Chinese Medicine, Guangdong Pharmaceutical University, Guangzhou, P.R. China

**Keywords:** *Jasminum lanceolaria*, chloroplast genome, phylogenetic analysis

## Abstract

*Jasminum lanceolaria* Roxb. (1996) is an important medicinal herb with diverse applications. It is native to China and belongs to the family Oleaceae. In this study, we reported the complete chloroplast genome sequence of *J. lanceolaria*. The assembled genome has a total length of 163,015 bp, with an overall GC content of 38.87%, and contained 133 genes, including 87 protein-coding genes, 38 tRNA genes, and eight rRNA genes. Phylogenetic analysis revealed that *J. lanceolaria* was closely related to *Jasminum polyanthum*. These findings provided valuable genomic resources and a reference for the taxonomic identification of *J. lanceolaria.*

## Introduction

*Jasminum lanceolaria* Roxb. (1820) is a species in the Oleaceae family (Quang et al. 2013), commonly known as the olive family, which comprised a diverse range of flowering plants, including economically important species such as olives (*Olea europaea*) and jasmine (*Jasminum*). The genus *Jasminum* consisted of shrubs and vines, and *J. lanceolaria* was characterized by its lance-shaped leaves and fragrant flowers (Rescigno et al. 2025). This climbing shrub typically occurs in low-altitude thickets from southeastern China to India (Balkrishna et al. 2021). Its stems and roots had traditionally been used to treat rheumatism and fever (Yan et al. 2015), while the leaves served as an anti-inflammatory agent (Sun et al. 2008).

The genus *Jasminum* contained a diverse array of bioactive compounds, including flavonoids (Sun et al. 2007), lignans, phenolic compounds, and glycosides (Jia-Ming et al. 2009), which exhibited antioxidant, anti-inflammatory, and antitumor activities (Tan et al. 2024). Despite the economic, medicinal, and ornamental importance of *J. lanceolaria*, its evolutionary relationships to other cogenera species in the *Jasminum* genus remained poorly understood. Chloroplasts are universal organelles found in plants. They have their own genomes. The relatively conserved structure, uniparental inheritance, and moderate mutation rates make the chloroplst genomes valuable molecular markers for phylogenetic reconstruction and species identification (Wang et al. 2021).

In the present study, we aimed to sequence and annotate the complete chloroplast genome of *J. lanceolaria*, thereby providing valuable data for phylogenetic analyses and lay the foundation for accurate identification of *J. lanceolaria* and products.

## Materials and methods

**Figure 1.**
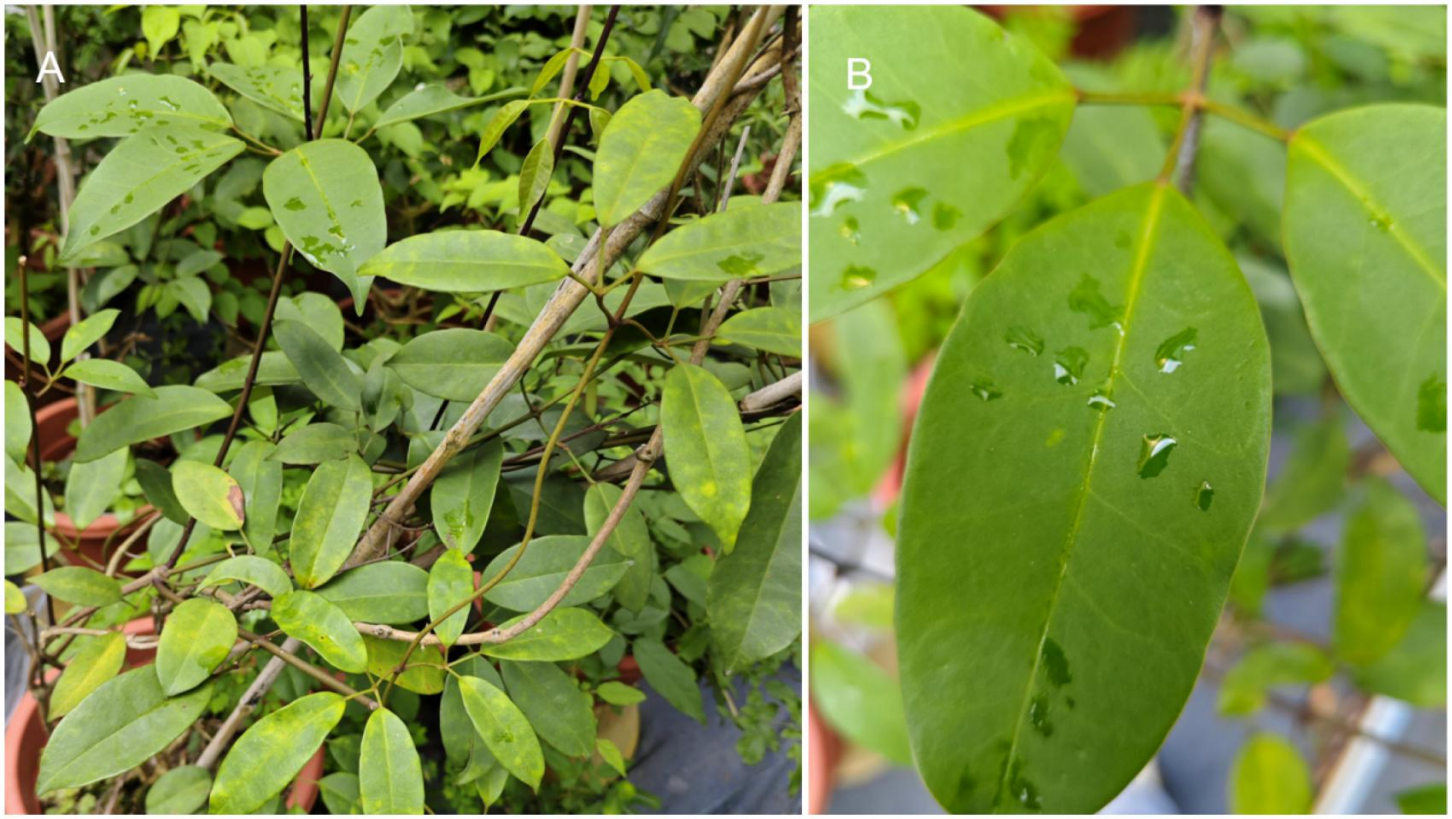
The Pictures of the individual plant of *J. lanceolaria*, photographed by guangfu Tian (tianguangfu@scbg.ac.cn) in South China Botanical Garden, Guangdong, China. (A) Growth form of *J. lanceolaria*, showing the scandent shrub with glabrous or pubescent stems, terete branchlets, glabrous petioles, opposite leaves, and a terminal leaflet with distinct petiolule. (B) Leaf morphology of *J. lanceolaria*, showing 3-foliolate leaves with elliptic, ovate, or lanceolate leaflets.

Fresh and healthy leaves of *J. lanceolaria* were collected from South China Botanical Garden, Guangdong Province, China (23°10′46.6″N, 113°21′6.7″E). Pictures of the plant are shown in Figure 1. The plant specimen was identified by GuangFu Tian (tianguangfu@scbg.ac.cn). The voucher specimen was deposited in the herbarium at the Institute of Medicinal Plant Development under the voucher number JXHC54 (Figure 1).

Total genomic DNA was isolated from *J. lanceolaria* leaf tissues using the Plant Genomic DNA Kit (Tiangen, Beijing, China) following the manufacturer’s protocol. DNA purity was assessed using a NanoDrop spectrophotometer, and DNA concentration was quantified using a Qubit fluorometer. Finally, the DNA integrity was evaluated by agarose gel electrophoresis.

The DNAs were subjected to library construction following the recommendation of the manufacturers (Illumina, USA). Sequencing was performed on a Illumina Xplus platform, generating paired-end reads of 150 bp with a total raw data volume of approximately 10 GB. The raw sequencing reads were processed for quality control by using FastQC for the evaluation overall sequencing quality. And Fastp v0.2 (Chen 2023) was used to remove adapter sequences and low-quality reads. The resulting clean data were then used for the de novo assembly of the complete chloroplast genome by using GetOrganelle v1.7.7.1 (Jin et al. 2020) with default parameters. The resulting circular genome was visualized by using Bandage v0.8.1 (Wick et al. 2015). To evaluate genome coverage, we mapped the sequencing reads back to the assembled chloroplast genome using BWA v0.7.17 (Li 2013), and calculated the coverage depth using Samtools v1.13 (Danecek et al. 2021). Genome annotation was performed by using CPGAVAS2 (Shi et al. 2019), and the annotation of tRNA genes was further verified with tRNAscan-SE v2.0 (Chan et al. 2021). To ensure the accuracy of the annotation, we manually corrected the start and stop codons as well as intron/exon boundaries using Geneious Prime 2025, with the chloroplast genome sequences of *Jasminum fluminense* (NC_042272.1) and *J. tortuosum* (NC_034691.1) serving as the reference sequences. The chloroplast genome map, along with the cis-splicing genes and the trans-splicing gene (*rps12*), was generated by using CPGView (Liu et al. 2023).

For phylogenetic analysis, chloroplast genome sequences from 29 additional species within Oleaceae were retrieved from the GenBank. Two species, *Callicarpa longissima* (NC_088747.1) and *Callicarpa macrophylla* (NC_058323.1), were used as outgroup species. Multiple sequence alignments of the complete chloroplast genome sequences were generated using MAFFT v7.490 (Katoh and Standley 2013). We used Modelfinder to determine the best model (https://www.nature.com/articles/nmeth.4285), which was found to be TVM+F + R5. Maximum likelihood (ML) trees were constructed by using IQ-TREE v2.3.3 (Lanfear et al. 2020), under the best-fit model, and node support was assessed with 1000 bootstrap replicates. The resulting phylogenetic tree was visualized by using the Interactive Tree of Life (iTOL) online tool (Letunic and Bork 2024) (https://itol.embl.de/). The complete chloroplast genome sequence of *J. lanceolaria* was submitted to the GenBank database and was assigned the accession number (PX412911).

## Results

Visualization of the assembled chloroplast genome of *J. lanceolaria* using Bandage indicated a complete and circular structure. The average sequencing depth was 4951.13×, with a minimum coverage of 1313×. The absence of uncovered regions indicated a high degree of assembly completeness and reliability (Figure S1).

The chloroplast genome of *J. lanceolaria* was 163,015 bp in length and has an overall GC content of 38.87%. It exhibits the typical quadripartite structure, consisting of a large single-copy (LSC) region of 90,557 bp, a small single-copy (SSC) region of 13,088 bp, and a pair of inverted repeat (IR) regions, each 29,685 bp in length.

**Figure 2.**
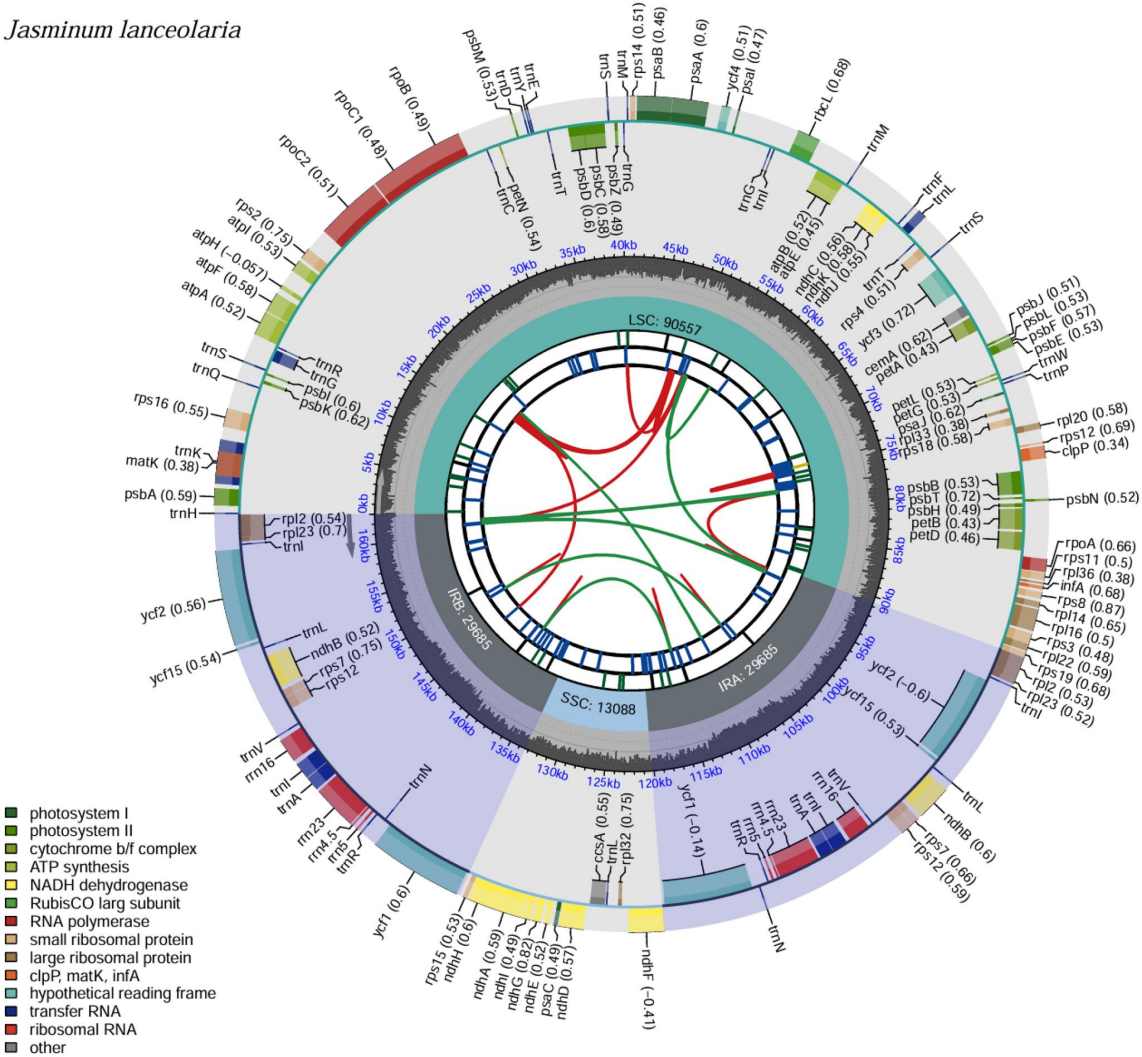
Schematic map illustrating the overall features of the chloroplast genome of *J. lanceolaria*. The map comprises six concentric tracks by default. From the center outward: the first track displays dispersed repeats; the second shows long tandem repeats as short blue bars; the third indicates short tandem repeats (microsatellites) as colored bars. The fourth track marks the structural regions of the genome, including SSC, LSC, IRa, and IRb. The fifth track plots the GC content across the genome. The sixth track presents annotated genes, with optional codon usage bias shown in parentheses after gene names. Genes are color-coded based on functional categories, as indicated in the legend at the bottom left. Genes transcribed on the inner and outer circles are oriented clockwise and counterclockwise, respectively.

The GC content varied across different regions: 35.75% in the LSC, 32.36% in the SSC, and 41.46% in the IRs. A total of 133 genes were annotated in the chloroplast genome, including 87 protein-coding genes, 38 transfer RNA (tRNA) genes, and 8 ribosomal RNA (rRNA) genes (Figure 2). Among the protein-coding genes, 10 were identified as cis-splicing genes, each containing a single intron (Figure S2). Additionally, *ycf3* gene contained two introns. The *rps12* gene was characterized as a trans-splicing gene (Figure S3), similar to those found in the chloroplast genomes of other plant species. The structure of these splicing genes serves as a quality indicator of the annotation. No unusual strucure found for these genes made us to concluded that the genome was annotated correctly.

**Figure 3.**
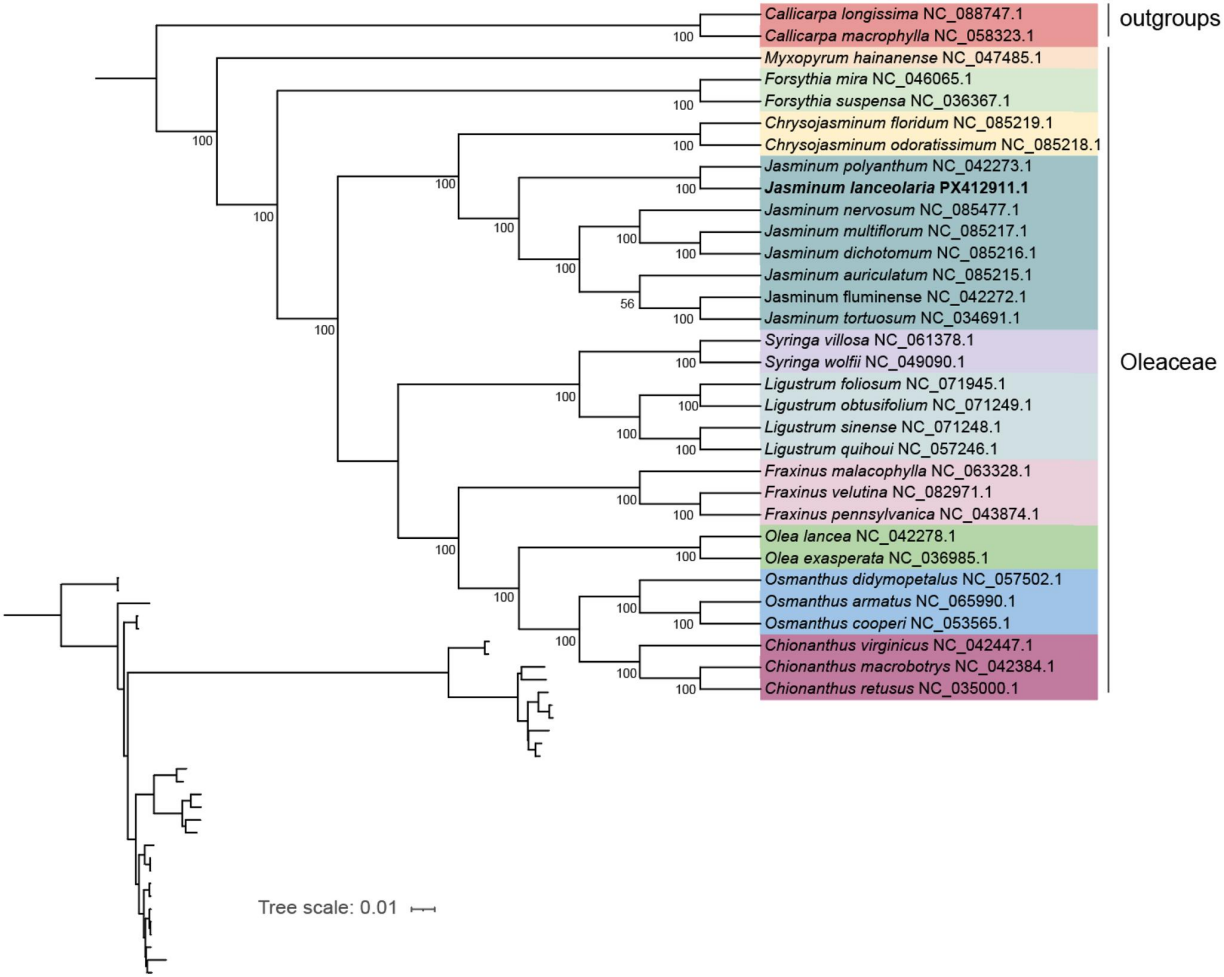
Phylogenetic tree of 29 additional species within oleaceae inferred using the ML method based on complete chloroplast genome sequence. *Callicarpa longissima* (NC_088747.1) and *Callicarpa macrophylla* (NC_058323.1) (Liu et al. 2022) was selected as outgroup. Numbers at each node represent the bootstrap values. The position of *J. lanceolaria* is marked in bold. The sequences used for constructing the phylogenetic tree are as follows: *Jasminum multiflorum* NC_085217.1 (Xu et al. 2024), *Jasminum fluminense* NC_042272.1, *Jasminum auriculatum* NC_085215.1(Xu et al. 2024), *Jasminum polyanthum* NC_042273.1, *Jasminum dichotomum* NC_085216.1, *Jasminum tortuosum* NC_034691.1, *Jasminum nervosum* NC_085477.1 (Le and Do 2024), *Chrysojasminum floridum* NC_085219.1, *Chrysojasminum odoratissimum* NC_085218.1, *Olea lancea* NC_042278.1, *Olea exasperata* NC_036985.1, *Syringa villosa* NC_061378.1, *Syringa oblata* NC_057990.1 (Yang et al. 2023), *Syringa wolfii* NC_049090.1 (Liu et al. 2020), *Fraxinus velutina* NC_082971.1, *Fraxinus malacophylla* NC_063328.1 (Duan et al. 2020), *Fraxinus pennsylvanica* NC_043874.1 (Yi et al. 2019), *Myxopyrum hainanense* NC_047485.1 (Zhu et al. 2020), *Forsythia mira* NC_046065.1 (Gao et al. 2019), *Forsythia suspensa* NC_036367.1 (Wang et al. 2017), *Chionanthus retusus* NC_035000.1 (He et al. 2017), *Chionanthus virginicus* NC_042447.1 (Wadl et al. 2022), *Chionanthus macrobotrys* NC_042384.1, *Osmanthus cooperi* NC_053565.1 (Wang et al. 2019), *Osmanthus didymopetalus* NC_057502.1 (Zhao et al. 2020), *Osmanthus armatus* NC_065990.1 (Du et al. 2019), *Ligustrum quihoui* NC_057246.1 (Wang et al. 2019), *Ligustrum obtusifolium* NC_071249.1 (Long et al. 2023), *Ligustrum sinense* NC_071248.1 (Long et al. 2023).

The phylogenetic analysis revealed that *J. lanceolaria* was more closely related to species in the genus *Chrysojasminum* at the intergeneric level, with strong bootstrap support values (Figure 3). At the intrageneric level, *J. lanceolaria* was most closely related to *Jasminum polyanthum* (NC_042273.1).

## Discussion and conclusion

Here, the chloroplast genome of *J. lanceolaria* was assembled and annotated for the first time, with a length of 163,015 bp and a total of 133 genes. This genome was comparable in size (159,404-165,352 bp) (Xu et al. 2024) to other *Jasminum* species, exhibiting a structure and gene composition that reflected the evolutionary conservation of plastid genomes within the genus.

Phylogenomic analyses based on complete chloroplast genomes yielded results that were largely consistent with previous phylogenies (Le and Do 2024; Xu et al. 2024). At the intergeneric level, *J. lanceolaria* was closely related to members of the genus *Chrysojasminum*, whereas within the *Jasminum* genus, it was clustered most closely with *J. polyanthum*. According to the most recent classification system of Oleaceae (Wallander and Albert 2000; Green 2004; The Angiosperm Phylogeny Group 2016), species within each genus formed a well-supported monophyletic clade, and the phylogenetic tree inferred from complete chloroplast genome sequences effectively distinguished species and reliably resolved their phylogenetic relationships.

To further refine the phylogenetic framework within *Jasminum*, future studies integrating nuclear genomic data, such as transcriptomes or low-copy nuclear genes, and broader taxon sampling will be valuable. Overall, the complete chloroplast genome of *J. lanceolaria* provided a valuable genomic resource for phylogenetic studies and offers important data for species identification.

## Supplementary Material

Supplemental Material

## Data Availability

The genome sequence data that support the findings of this study are publicly available from the NCBI GenBank database under accession number PX412911. The associated BioProject, BioSample, and SRA accession numbers are PRJNA1332946, SAMN51784821, and SRR35567767, respectively.
