# Supplementary material for: The complete chloroplast genome and phylogenetic analysis of Jasminum lanceolaria Roxb. (Oleaceae)
Source: Mitochondrial DNA B Resour. 2026 Feb 4;11(3):367–72. doi: 10.1080/23802359.2026.2622811 (PMC12875101; doi:10.1080/23802359.2026.2622811)

Figure S1. Overall coverage and sequencing depth of the *J. lanceolaria* chloroplast genome assembly. The X-axis represents genome position, and the Y-axis shows sequencing depth at each base.


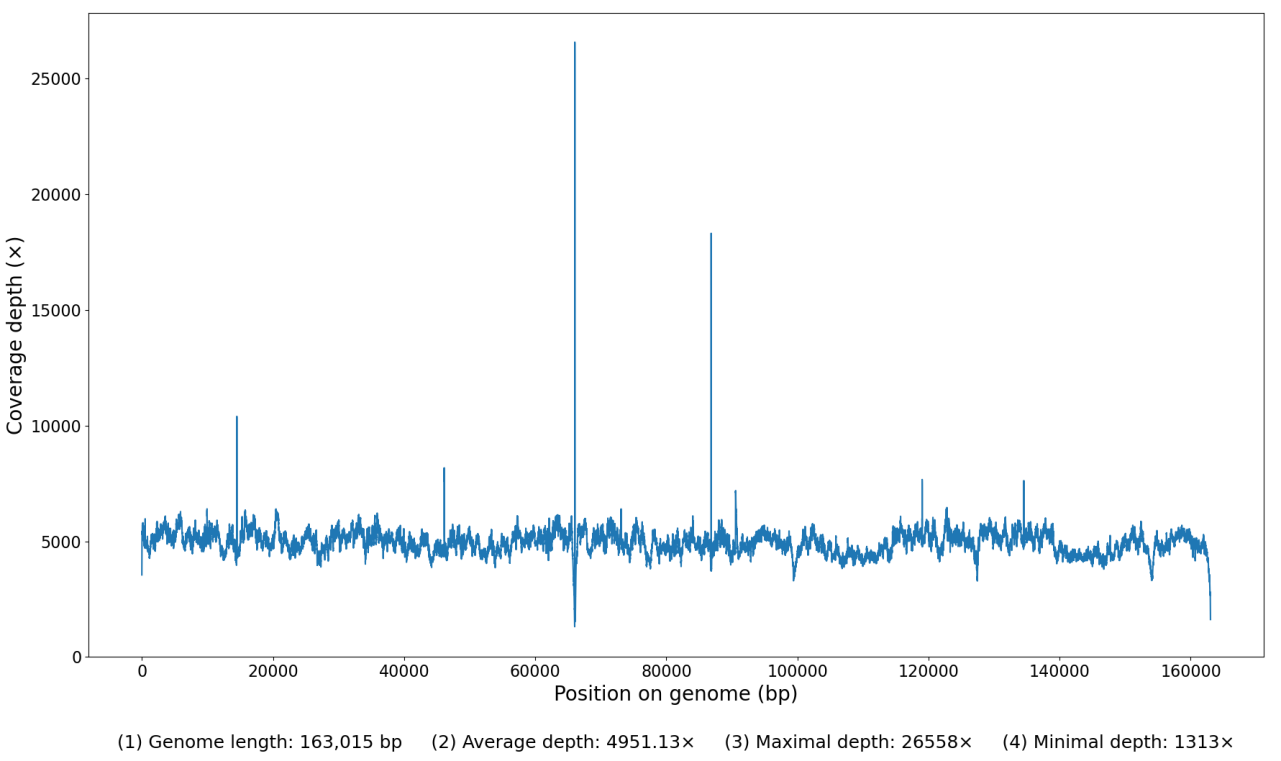


Figure S2. Schematic map of cis-spliced genes in the *J. lanceolaria* chloroplast genome. Genes are arranged vertically from top to bottom according to their positions in the chloroplast genome. Gene names are shown on the left, with gene structures depicted on the right. Exons are shown in black, introns in white, and arrows indicate the direction of gene transcription (sense strand). Exon and intron lengths are not drawn to scale and serve only to illustrate the gene structure.


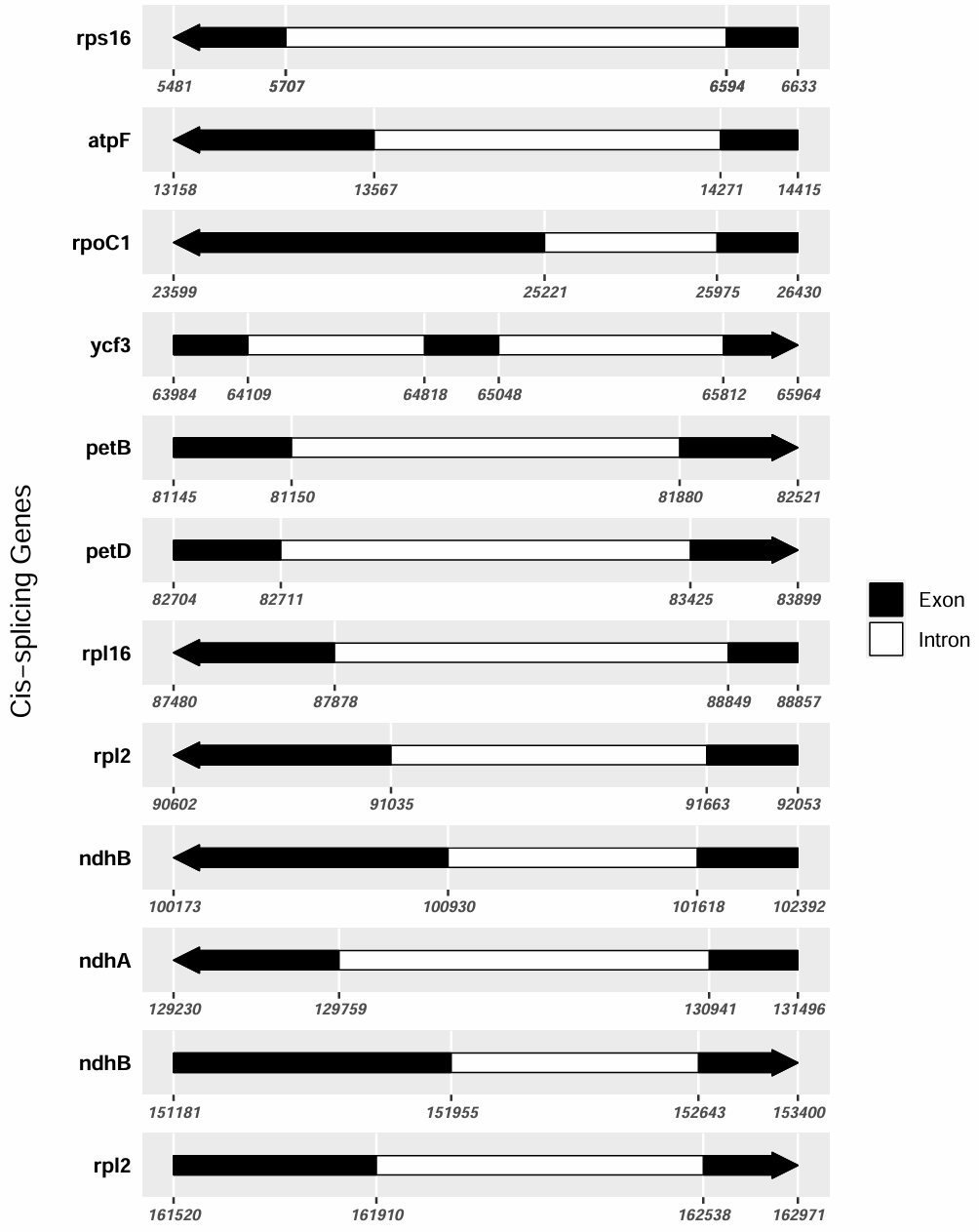


Figure S3. Schematic map of the trans-spliced gene *rps*12 in the *J. lanceolaria* chloroplast genome. The gene consists of three unique exons, two of which are duplicated due to their location within the IR regions


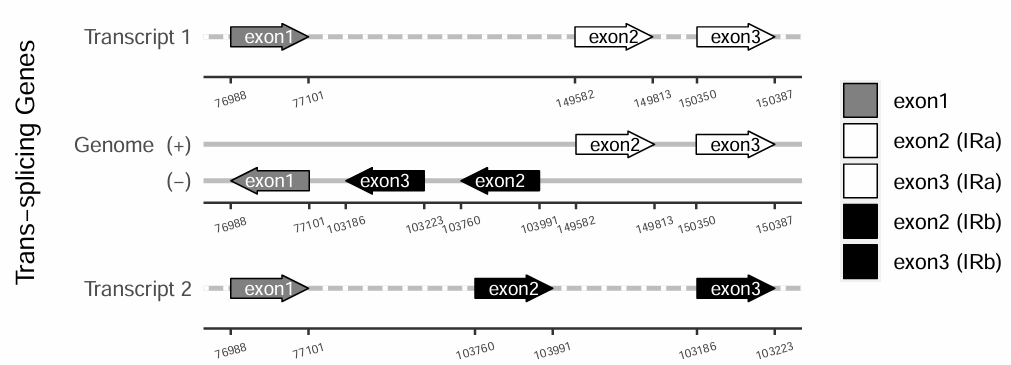

Supplement: Supplemental Material [file TMDN_A_2622811_SM0043.docx]
